# Spatial distribution of rural population from a climate perspective: Evidence from Jiangxi Province in China

**DOI:** 10.1371/journal.pone.0248078

**Published:** 2021-03-04

**Authors:** Liguo Zhang, Xi Lin, Langping Leng, Yongming Zeng

**Affiliations:** School of Economics, Jiangxi University of Finance and Economics, Jiangxi, China; Northeastern University (Shenyang China), CHINA

## Abstract

The research on rural population distribution from a climate perspective is rare. Therefore, this study adopts this perspective and uses the ordinary least squares and spatial econometric models to explore the spatial distribution characteristics of the rural population in the Poyang Lake ecological economic zone. Results show that (1) a significant spatial autocorrelation is present in the distribution of rural population, and a spatial correlation exists between the population distribution and climatic factors, (2) the influence of climatic factors on the distribution of rural population in the Poyang Lake ecological economic zone is greater than that of economic factors, and (3) the annual average sunshine and annual average rainfall have a significant negative effect on the distribution of the regional rural population, which is contrary to the expectations., so we then analyze this negative effect on the regional rural population distribution. It is found that (1) the influence of climate factors on the distribution of rural population in lake area is far more than that of economic factors, and more consideration should be given to the influence of climate factors on the population distribution in the lake area, (2) different geographical capital and natural resource endowment, the influence of climate on micro-regional population distribution may be different from the general law, (3) the spatial measurement model which takes spatial dependence into account can reveal the influence of climate on rural population distribution more accurately.

## Introduction

Population distribution has always been a research hotspot in the field of population studies. Given the acceleration of urbanization and the imbalance of regional economic development, the current climate environment deteriorates, and natural disasters frequently occur. As a result, the population distribution pattern is constantly reshaped. Related studies on population distribution gradually attract the attention of scholars and governments. Many research on population distribution exist [1–4], especially in the early “heavy urban, light rural” background [5]. However, most of these studies focused on urban population distribution, and few explored the rural population distribution. The change in China’s rural population profoundly affects the social economy, thereby becoming the main contradiction of the rural population development and basic national conditions [6]. The report of the 19th National Congress of the Communist Party of China proposed the implementation of rural revitalization to fundamentally solve the “problems about agriculture, rural, and peasantry,” as well as the unbalanced and inadequate problems of rural development, because the rural population is an important factor that affects the process and effectiveness of rural revitalization.

The study on rural population distribution is mainly based on urbanization and hollowing [7, 8]. However, investigating such a distribution on the basis of climate perspective is rare. The relationship between population and climate change lacks systematic research [9]. The existing population distribution research is mainly focused on spatial and temporal distributions and evolution [10, 11], coupling analysis with economic or geographical factors, and fitness research [12–14]. According to the research, the lake area is a typical and sensitive area of global climate change, and the inland lake area is the indicator of climate change in the lake area [15]. Poyang Lake is the largest freshwater lake in inland China and the second largest lake in inland China which plays a great role in conserving water and improving local climate and environment, so it is the most important wetland. This paper takes Poyang Lake Ecological Economic Zone as the research area. On the one hand, Poyang Lake Ecological Economic Zone is a sensitive area to deal with climate change, on the other hand, rural population is a sensitive group to deal with climate change, which is more representative to study the influence of climate change in sensitive areas on the spatial distribution of sensitive groups. The selection of Poyang Lake Ecological Economic Zone as the research area has certain reference significance for the rational distribution of rural population in lake area under the background of global climate deterioration.

This study selects the rural population of the Poyang Lake ecological economic zone as the research object and takes the county (city, district) as the research scale. In addition, the spatial autocorrelation and spatial dependence of population distribution are comprehensively considered. The influence of climatic factors on rural population distribution is analyzed using the spatial measurement method to understand the distribution law of rural population from a climate perspective.

## Data sources and research methods

### Data sources

The data used in this paper are gathered from three aspects. First, the related topographic data of the Poyang Lake ecological economic zone are derived from National Earth System Science Data Center and extracted from the shapefile format map by using the statistical tools in the ArcGis software. Second, the relevant climate data, including temperature, rainfall, sunshine and wind speed, are extracted from National Meteorological Information Center and 839 meteorological stations in China by spatial interpolation of data. Third, the other social and economic factors are obtained from the statistical yearbook of prefecture-level cities in Jiangxi Province.

### Research method

#### Spatial autocorrelation analysis

Spatial autocorrelation refers to the potential interdependence among the observation data of several variables in the same distribution area. According to Tobler’s First Law, the phenomenon of spatial correlation is universal, and the correlation of close places is stronger than that of far ones [16]. Spatial autocorrelation is typically used to detect the spatial correlation and cluster of some variables, which is known referred to as spatial dependence. Compared with the traditional autocorrelation analysis, spatial autocorrelation introduces a spatial weight matrix to represent the degree of spatial correlation among regions. The spatial weight matrix is determined by the adjacency of the spatial unit or the distance among these units. Spatial autocorrelation can be divided into two categories: global and local spatial autocorrelation [17].

#### Global spatial autocorrelation

Global spatial autocorrelation is used to analyze whether cluster characteristics are present in the entire spatial range of geographical data. However, this category cannot accurately identify the specific cluster area. Global Moran’s I is a commonly used global spatial autocorrelation statistical index that is defined as

$$I\;=\;\frac{n\sum_{i\;=\;1}^{n}\sum_{j\neq i}^{n}w_{ij}(x_{i}-\overset{-}{x})(x_{j}-\overset{-}{x})}{\sum_{i\;=\;1}^{n}\sum_{j\neq i}^{n}w_{ij}\sum_{i\;=\;1}^{n}{\left(x_{i}-\overset{-}{x}\right)}^{2}}\;=\;\frac{\sum_{i\;=\;1}^{n}\sum_{j\neq i}^{n}w_{ij}(x_{i}-\overset{-}{x})(x_{j}-\overset{-}{x})}{S^{2}\sum_{i\;=\;1}^{n}\sum_{j\neq i}^{n}w_{ij}},$$ (1)

where *n* represents the total number of the studied regional space units, *w*_*ij*_ is the spatial weight matrix, *x*_*i*_ and *x*_*j*_ denote the attribute values of space units *i* and *j*, respectively, and $\overset{-}{x}$ is the average value of all attribute values for indicators. The value of Global Moran’s I ranges from −1 to 1; the closer the value is to 1 (i.e., greater than 0), the stronger the positive correlation is, that is, a cluster of spatial units with the same attributes (high and high adjacent or low and low adjacent). By contrast, the closer the value is to −1 (i.e., less than 0), the stronger the negative correlation is, that is, a cluster of spatial units with different attributes (the high value is adjacent to the low value). If the value is close to 0, the spatial units are irrelevant.

#### Local spatial autocorrelation

To study the heterogeneity of spatial autocorrelation, local spatial autocorrelation is typically used to test whether a cluster area is present in the local spatial units. This exploration makes up for the limitation of the global spatial autocorrelation, which cannot reflect local aggregation. The local Moran’s I index (i.e., local indicator of spatial association [LISA]) is proposed to measure the correlation between the spatial unit and its surrounding spatial units [18]. On the basis of this index, the Moran scatter plot can be constructed to study the local spatial heterogeneity. The scatter plot includes a Cartesian coordinate system, where the abscissa represents ***Z***_***i***_, **∑*w***_***ij***_***Z***_***j***_ denotes the normalized value of the central target unit, and the ordinate is the space lag value.

In accordance with the attributes of the Cartesian coordinate system, four types of local spatial correlation can be obtained.

$$\{\begin{array}{c} Z_{i}>0,\sum W_{ij}Z_{j}>0(+,+),first\;quadrant,High-High\;Cluster\;(H-H)\\ Z_{i}<0,\sum W_{ij}Z_{j}>0(-,+),second\;quadrant,Low-High\;Cluster\;(L-H)\\ Z_{i}<0,\sum W_{ij}Z_{j}<0(-,-),third\;quadrant,Low-Low\;Cluster\;(L-L)\\ Z_{i}>0,\sum W_{ij}Z_{j}<0(+,-),forth\;quadrant,High-Low\;Cluster\;(H-L) \end{array}\;$$ (2)

where the High–High Cluster indicates that the central region is the same as the adjacent region, and the attribute value is high; the Low–Low Cluster suggests that the central region is the same as the adjacent region, but the attribute value is low; the High–Low Cluster indicates that the attribute value of the central region is high, whereas that of the adjacent region is low; and the Low–High Cluster implies that the attribute value of the central region is low, whereas that of the adjacent region is high.

#### Spatial constant coefficient regression model

Compared with the ordinary least squares (OLS) model, the spatial constant coefficient regression model adds autocorrelation factors, and the spatial dependence and spatial weight are considered. The spatial constant coefficient regression model includes the spatial lag model (SLM) and spatial error model (SEM), which are estimated through the maximum likelihood method [19].

1. SLM
  SLM is used to explore the spatial dependence caused by spatial diffusion and spatial spillover effects. A spatial autocorrelation is assumed to be present among the dependent variables in the study area and absent among the independent variables. This model can be mathematically expressed as
  $$Y\;=\;\rho W_{1}Y+\beta X+\varepsilon$$
  $$\varepsilon \sim N(0,\sigma^{2}I_{n}),$$ (3)
  where *Y* is a vector with a dimension of *n* × 1, *X* is an independent variable matrix with a dimension of *n* × *k*, *W*_1_ is a spatial weight matrix with a dimension of n × n, *ρ* represents spatial autocorrelation coefficient, *β*represents the independent variables coefficient of *k* × 1 dimension, *ε* represents random error items, *σ*^2^ is the variance of *ε*.
2. SEM
  SEM assumes that the error has a spatial autocorrelation. This model is defined as
  Y=βX+ξ
  $$\xi \;=\;\lambda W_{2}\xi +\varepsilon ,$$ (4)
  where *Y* is a residual vector with a dimension of *n* × 1, *λ* represents the residual correlation coefficient, *W*_2_ is a residual vector with a dimension of *n* × *n*, and the other parameters are the same as those of SLM.

## Spatial characteristics of the rural population distribution in the Poyang Lake ecological economic zone

### Overview of the Poyang Lake ecological economic zone

**Fig 1:**
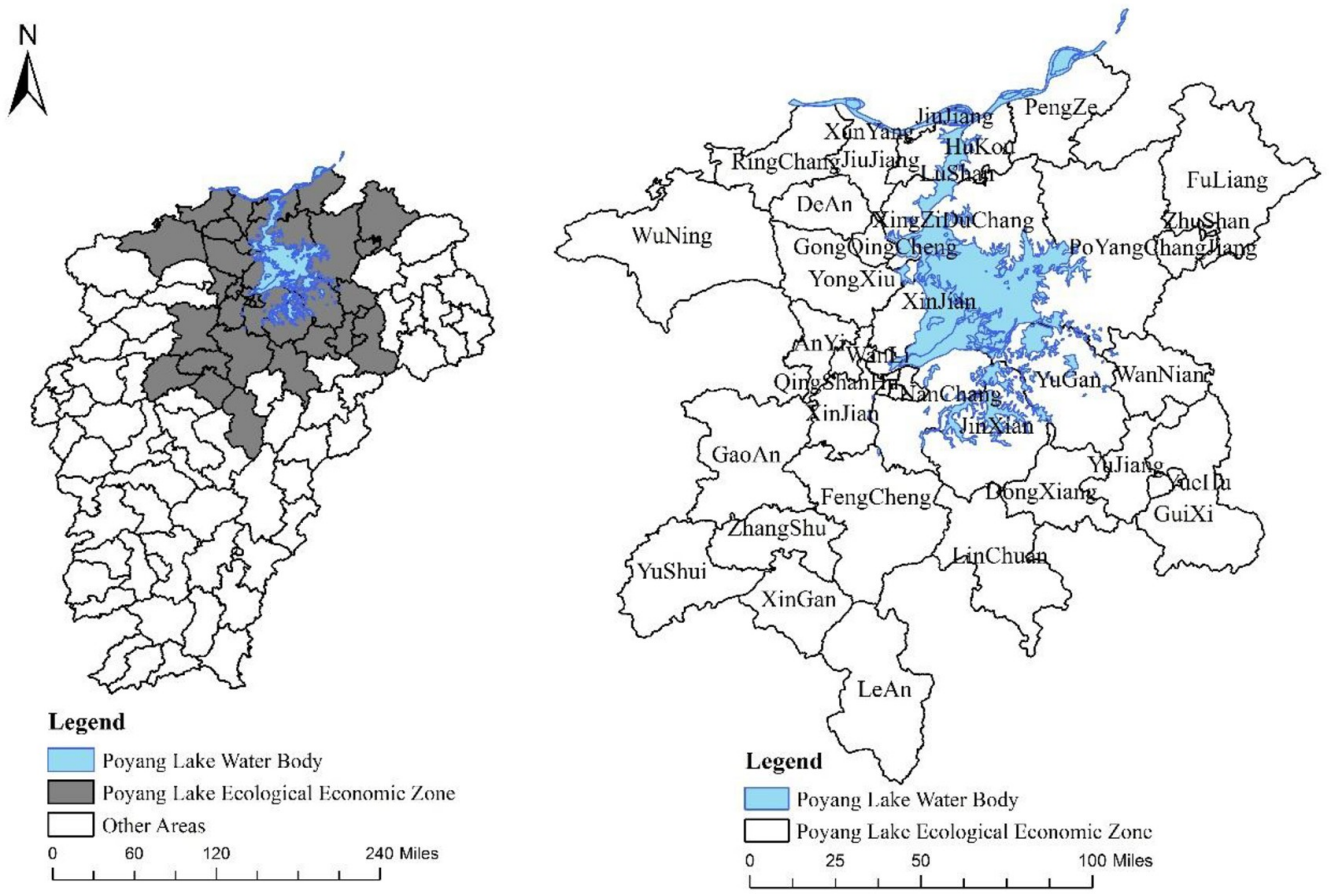
Geographical location diagram and administrative plan.

The Poyang Lake ecological economic zone is located in the middle and lower reaches of the Yangtze River and northern part of Jiangxi Province. This area is a special economic zone whose core is the Poyang Lake (114°29’ E~117°42’ E, 27°30’ N) and important strategic concepts include protecting the ecology and developing the economy (Fig 1). The total area is 51200 km^2^, which account for 30.68% of the total area of the province. The scope of this zone includes 38 counties (cities, districts) under the jurisdiction of Nanchang, Jiujiang, Shangrao, and other urban areas. By the end of 2016, all counties (cities and districts) under the jurisdiction of the Poyang Lake ecological economic zone had a total population of 20.581 million and a rural population of 10.652 million, which accounted for roughly 51.6% of the total population. The poor population was 308600, which is approximately 2.9% of the rural population.

### Spatial distribution characteristics of the rural population by county

**Fig 2:**
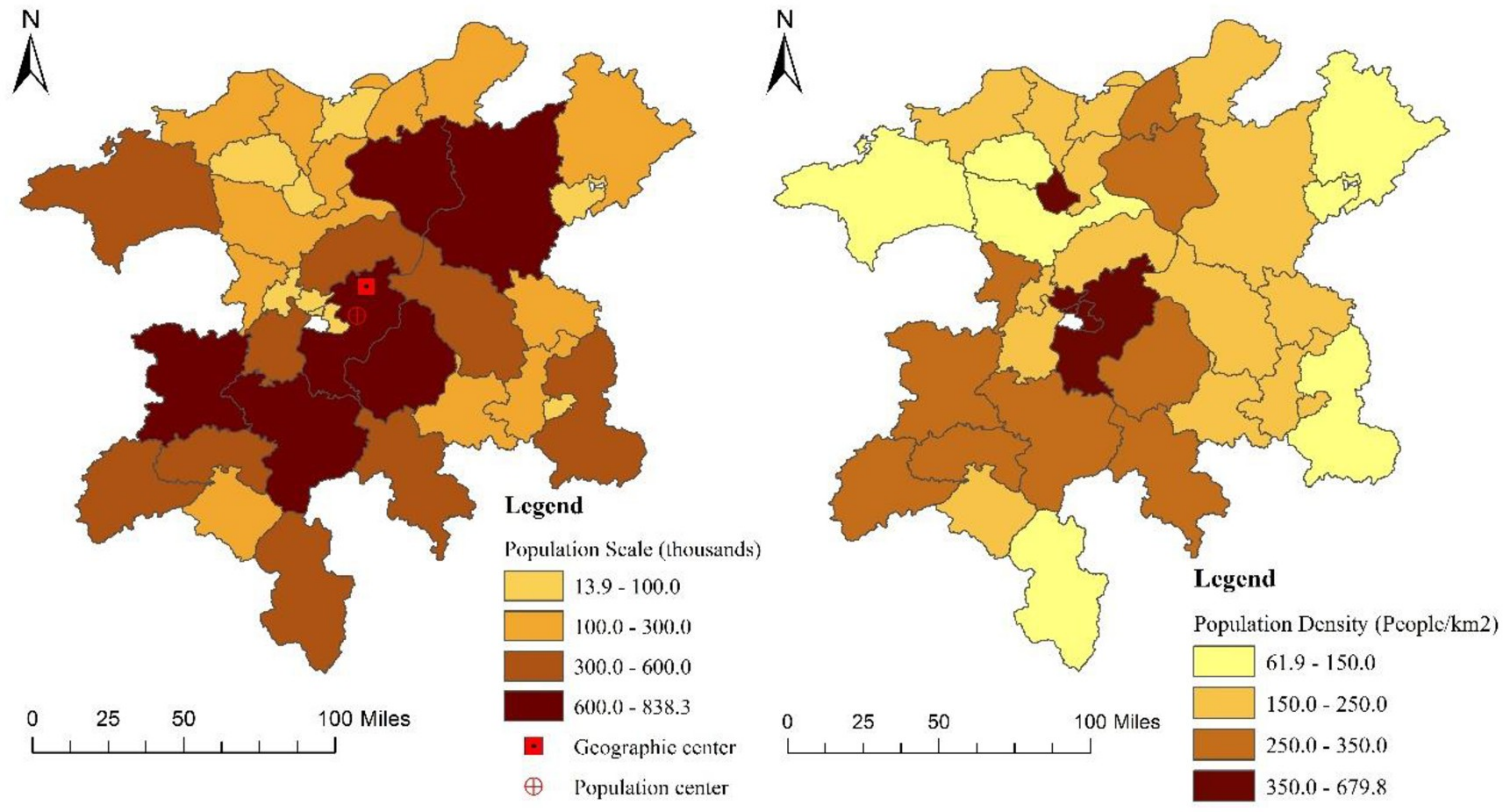
Spatial distribution of county population size and density.

As previously mentioned, 38 counties (cities and districts) are incuded in the Poyang Lake Poyang Lake ecological economic zone. However, only 34 spatial analysis units are considered in this study because the urbanization rates of the Xunyang District, Zhushan District, Xihu District, and Qingyun Spectrum reach 100%, and these areas have no rural population distribution. The data about population size and density in Fig 2 are obtained from the statistical yearbook of prefecture-level cities in Jiangxi Province. As shown in Fig 2, substantial differences are present in the rural population scale and rural population density in the Poyang Lake ecological economic zone. Fengcheng City had the largest rural population (838200 people), whereas Yuehu District had only 13900 residents; the population of the former was approximately 60 times that of the latter. Gongqing City had the highest rural population density (679.8 people per square kilometer), whereas Fuliang County had the lowest (61.9 people per square kilometer); the population density of the former was roughly 11 times that of the latter.

The spatial distribution of rural population in the Poyang Lake ecological economic zone can be described as follows. First, the rural population in the central region is larger than that in the peripheral areas. Second, the central and southwest regions have high rural population density cluster areas, whereas the northeast region has a relatively small rural population density and is a low rural population density cluster area. Lastly, the rural population in the southwest part of the geographical center is slightly higher than that in the northeast part. The geographical center of the entire region is located at 116.129° E, 28.817° N, and the population center is located at 116.084° E and 28.677° N. Both points are located in the northeast part of Nanchang County. However, the population center deviates from the geographical center to the southwest, thereby indicating that the rural population distribution in the southwest region is relatively high.

## Spatial autocorrelation analysis of the rural population

### Global spatial autocorrelation analysis of rural population in county

**Fig 3:**
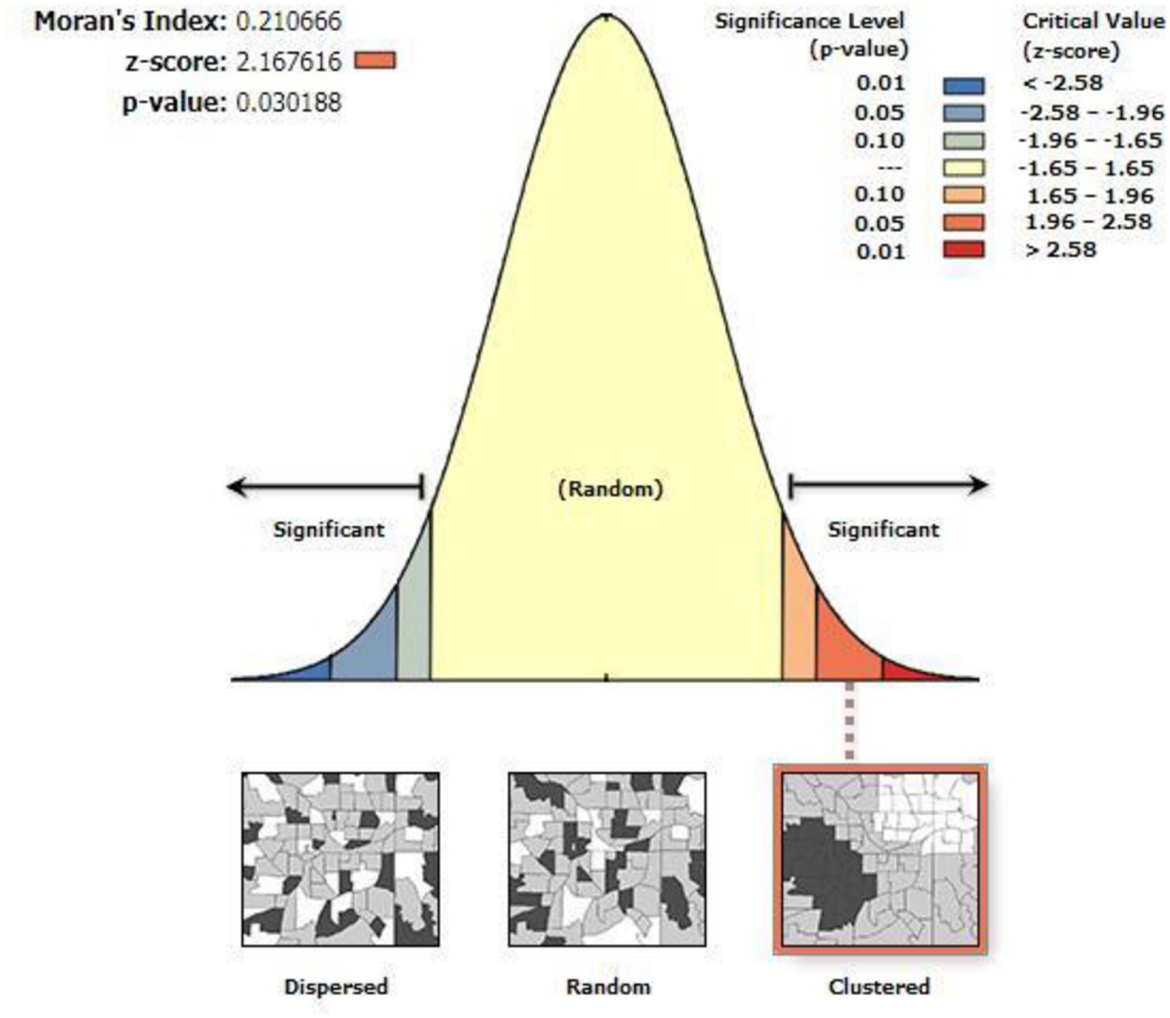
Moran’s I results of the distribution of rural population in the county.

The global spatial autocorrelation analysis of the rural population density in the Poyang Lake ecological economic zone is performed using ArcGis 10.1. The results are illustrated in Fig 3. Moran’s I index is 0.211, and the z value is 2.167, which satisfies the significance test at a level of 0.05. This result shows that a certain degree of spatial positive autocorrelation is present in the distribution of rural population in the Poyang Lake ecological economic zone, and the density of rural population in each region is not independent of the spatial distribution but presents a certain spatial cluster.

### Local spatial autocorrelation between climatic factors and rural population

**Fig 4:**
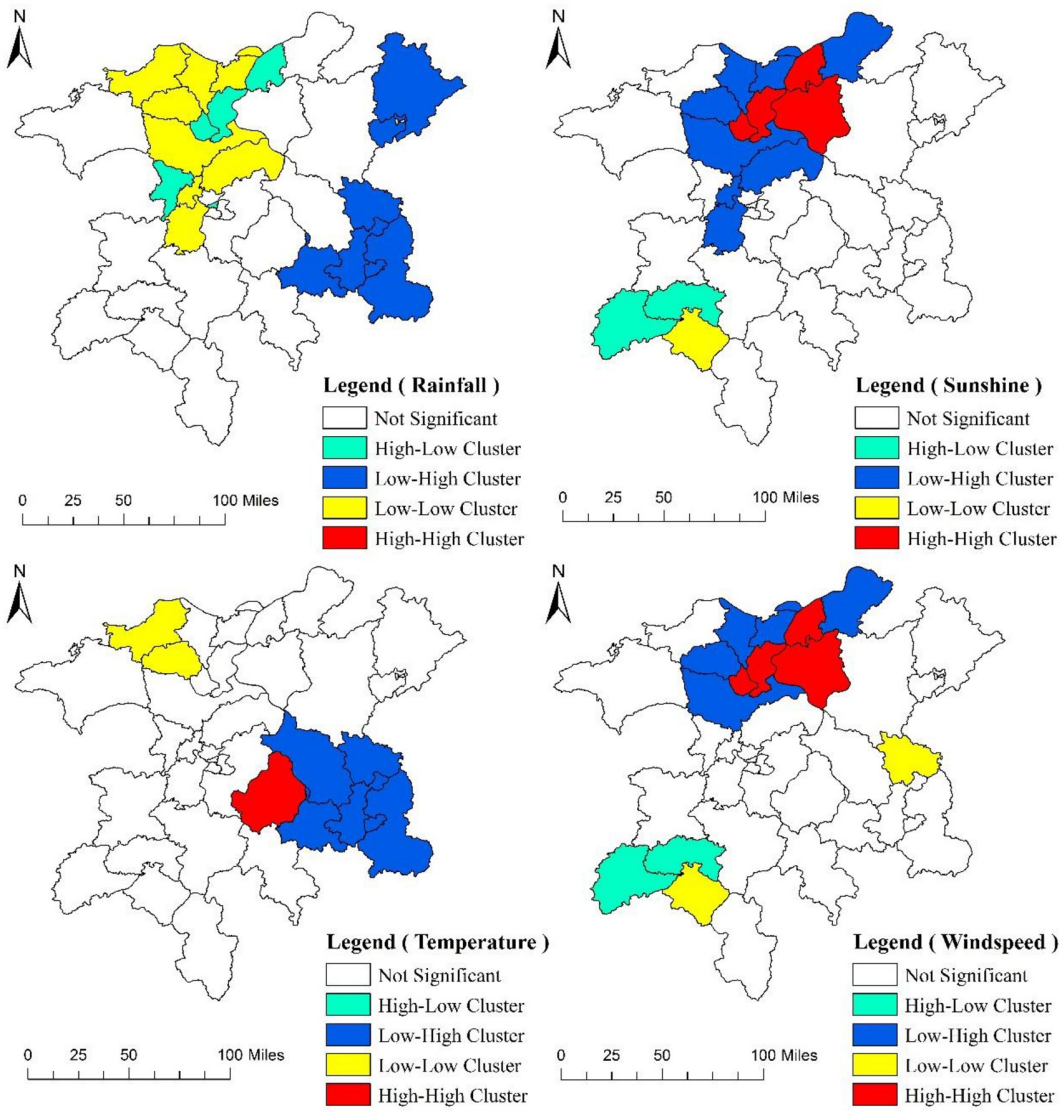
LISA cluster map of population density and climatic factors.

On the basis of the existing studies and considering the availability of data, four climate indicators are selected in this study, namely, annual average rainfall (AR), annual average sunshine (AS), annual average temperature (AT), and annual average windspeed (AW) [20–24]. The local spatial autocorrelation analysis of the rural population density and regional climatic factors in the Poyang Lake ecological economic zone is performed using the Geoda software, and the results are shown in Fig 4. From the perspective of AR, no High–High Cluster areas are present, and the northwest region presents the characteristics of a Low–Low (the rural population density in the central region, as well as the per capita rainfall in the surrounding areas, is small) and High–Low Clusters. Meanwhile, the eastern region mainly presents the characteristics of a Low–High Cluster. On the basis of AS, the High–High Cluster areas are concentrated in the northern region, the Low–High Cluster areas are distributed around the High–High Cluster areas, and the Low–Low and High–Low Cluster areas are distributed in the southwest region. From the perspective of AT, no High–Low Cluster areas exist, only one area (Jinxian County) presents the characteristics of a High–High Cluster, Low–Low Cluster areas are distributed in the northwest, and Low–High Cluster areas are mainly distributed in the southeast. In terms of the AW, the High–High Cluster areas are concentrated in the northern region, the Low–High Cluster areas are distributed around the High–High Cluster areas, the High–Low Cluster areas are located in the southwest, and the Low–Low Cluster areas, namely, Yushui and Wannian Counties, are respectively located in the southwest and the east regions.

The numbers of regions with significant local spatial autocorrelation between rural population distribution and AR, AS, AT, and AW are 19, 14, 9, and 13, respectively. The number of detailed cluster types is summarized in Table 1. The results show that a certain degree of local spatial autocorrelation exists between the distribution of rural population and various climatic factors.

**Table 1:** Spatial cluster of the rural population distribution and climatic factors.

Types	Climate Indicators			
	AR	AS	AT	AW
**High–High Cluster**	0	4	1	4
**Low–Low Cluster**	7	1	2	2
**Low–High Cluster**	7	7	6	5
**High–Low Cluster**	5	2	0	2
**Non-significant Regions**	15	20	25	21
**Total**	34	34	34	34

The results of the spatial autocorrelation analysis suggest that a positive spatial autocorrelation is present in the distribution of rural population in the ecological economic zone of Poyang Lake, and a certain degree of spatial correlation exists between the density of rural population and various climatic factors.

## Empirical analysis of rural population distribution in the Poyang Lake ecological economic zone

### Selection of variables

#### Selection of the dependent variable

Population density is a direct representation and an important index of population distribution. This factor is the most commonly used index in social science research and the most recognized index in demography. Therefore, this study selects the county’s rural population density of the Poyang Lake ecological economic zone as an index for measuring the distribution of rural population [25].

#### Selection of the independent variables

Numerous factors affect the population distribution. In accordance with the requirement of this study, the influencing factors of rural population distribution are divided into two categories: climatic and other factors. The climatic factors include the AR, AS, AT, and AW. The values of ALT, AGR, ELE, and DI are selected in accordance with the existing studies and by comprehensively considering the situation of the Poyang Lake ecological economic zone [26–30]. The specific indicators are listed in Table 2.

**Table 2:** Index system of the rural population distribution.

Variables		Index	Abbreviation	Unit
Dependent Variable		Rural population density	RPD	people/square kilometer
**Independent Variables**	**Climatic Factors**	Annual Average rainfall	AR	millimeter
		Annual Average Sunshine	AS	hour
		Annual Average Temperature	AT	centigrade
		Annual Average Windspeed	AW	meter/second
	**Other Factors**	Average Altitude	ALT	meter
		Output value of agriculture, forestry, animal husbandry, and fishery per unit area	AGR	10^4^ RMB/ hectare
		Rural electricity consumption per unit area	ELE	kilowatt-hour/square kilometer
		Per capita disposable income of rural residents	DI	RMB/people

### OLS model and results analysis

#### Model building

To overcome the problem of heteroscedasticity in cross-sectional data, this study analyzes all variables logarithmically in accordance with the general method of econometrics (same treatment with SLM and SEM). Disregarding the spatial dependence, the density of rural population is considered as the dependent variable, and the OLS model is constructed using the Stata 14 software. The influence direction and degree of the independent variables on the density of rural population are also analyzed.

The final result is displayed in Table 3. During the process of stepwise regression, AT fails to satisfy the significance test, and thus is eliminated. The constant terms and seven independent variables passed the significance test; the goodness of fit and adjusted goodness of fit of the model are 0.8480 and 0.8071, respectively. From the result of the multiple collinearity test, the variance inflation factor (VIF) of all independent variables is less than 10, and no multiple collinearity is observed. The probability of the Breusch–Pagan heteroscedasticity test is 0.3265, which satisfies the significant test at 1% level and indicates the absence of heteroscedasticity. In conclusion, the fitting effect of the OLS model is satisfactory.

**Table 3:** OLS model parameter estimation and test results.

Variables	Coefficient	t-statistic	p-value	VIF
**C**	98.5615***	4.9380	0.0000	
**lnAR**	−3.0027**	−2.6439	0.0137	1.6900
**lnAS**	−9.2576***	−3.1546	0.0040	4.5500
**lnAW**	0.8284**	2.1800	0.0385	4.3700
**lnALT**	−0.2530**	−2.6852	0.0125	4.0200
**lnAGR**	0.3093***	3.3395	0.0025	1.9100
**lnELE**	0.2435***	3.2372	0.0033	2.6900
**lnDI**	−0.3742*	−1.7552	0.0910	1.3900
**R**^**2**^	0.848		LogL	6.5306
**R**^**2**^**adj**	0.8071		AIC	2.9389
**P**_**bp**_	0.3265		SC	15.1498

**Note**: The symbols *, **, and *** indicate significance at levels of 10%, 5%, and 1%, respectively.

#### OLS results analysis

The south area experiences large amounts of sunshine and rainfall. The larger the sunshine and the higher the amount of rainfall, the greater the density of rural population. However, the results of the OLS analysis show that the AS and AR coefficients are negative, and the remaining positive and negative coefficients are consistent with the expected results. Given that the OLS model does not consider the spatial dependence, the credibility of the influence of the correlation coefficient on the regional rural population should be further explored. The spatial econometric models (SLM and SEM) must be constructed, and the optimal model should be selected before drawing conclusions and interpretations.

Spatial econometric model (SLM and SEM) and analysis of the optimal model results.

#### Model construction and optimal model determination

On the basis of the OLS model, SLM and SEM are constructed using the Geoda software, and the weight with the spatial inverse distance is set. Table 4 indicates that the probabilities of the Breusch–Pagan heteroscedasticity test for SLM and SEM are 0.2604 and 0.3265, respectively. Both models satisfiy the significant test at 1% level, thereby indicating that no heteroscedasticity is present between them. Similarly, the constant terms and independent variables of the two models pass the significance test.

**Table 4:** Parameter estimation and test results of the spatial econometric models.

Variables	SLM			SEM		
	Coefficient	Z-statistic	p-value	Coefficient	Z-statistic	p-value
**C**	95.6372***	5.1942	0.0000	92.4114***	6.4822	0.0000
**lnAR**	−2.5664**	−2.2779	0.0227	−3.2294***	−4.2519	0.0000
**lnAS**	−9.3730***	−3.6718	0.0002	−8.2041***	−3.7000	0.0001
**lnAW**	0.8532***	2.5881	0.0097	0.6881**	2.5312	0.0113
**lnAlt**	−0.2501***	−3.0590	0.0022	−0.2754***	−3.4294	0.0006
**lnAGR**	0.3028***	3.7414	0.0002	0.3013***	3.6106	0.0003
**lnELE**	0.2418***	3.6989	0.0002	0.2370***	3.5003	0.0005
**lnDI**	−0.3785**	−2.0464	0.0407	−0.3482**	−2.0240	0.0430
**R**^**2**^	0.8505			0.8601		
**P**_**bp**_	0.3577			0.3133		
**LogL**	6.7650			7.2253		
**AIC**	4.4699			1.5494		
**SC**	18.2072			13.7603		

**Note**: The symbols *, **, *** indicate significance at levels of 10%, 5%, and 1% respectively.

OLS model, SLM, and SEM are highly appropriate for analyzing the rural population distribution in the Poyang Lake. The rules of likelihood (LogL), Akaike information criterion (AIC), and Schwartz criterion (SC) are generally used to discriminate the optimal model. The larger the LogL and the smaller the AIC and SC, the better the model effect is [31]. The LogL of SEM is larger than those of SLM and OLS model, whereas the AIC and SC of the former are smaller than those of the latter two. This finding reveals that SEM is more satisfactory than the other two. The overall significance level of the SEM coefficient is higher than those of SLM and OLS model. The results suggest that SEM is the optimal model.

#### Analysis of the optimal model (SEM) results

By comparing the standard coefficients of each variable of the SEM, the degree of influence on the dependent variables from large to small is ranked as follows: AS, AR, AW, DI, AGR, ALT, and ELE. Given the logarithmic analysis of all variables, the coefficient represents the elasticity. The elasticity of the climatic factors is larger than that of the economic factors. This result indicates that the influence of the former on the distribution of rural population is greater than that of the latter. Generally speaking, economic factors play a leading role in the distribution of total population or urban population, while climate factors are more dominant than economic factors in the distribution of rural population. This also confirms that rural population is a vulnerable group to cope with climate change. The elasticity of the average elevation is small because the Poyang Lake ecological economic zone is mainly composed of plains. In addition, the negative influence of topographic factors, such as altitude, on the distribution of rural population in the region is small. The result of the comparison of the coefficients (absolute value) of the OLS model and SLM shows that in terms of climatic factors, the former underestimates the influence of rainfall on the distribution of rural population, overestimates the influence of sunshine and windspeed, and the values of the coefficient of other factors are not significantly different. According to the first theorem of geography, everything is related to everything else, but near things are more related to each other. This is especially true of climate factors, which have spatial correlation and spatial dependence in spatial distribution. The spatial econometric model considering spatial dependence can more accurately reflect the influence of climate factors on the distribution of rural population.

Moreover, all positive and negative signs corresponding to the OLS model and SEM are consistent. Therefore, the negative effects of rainfall and sunshine on the distribution of the regional rural population are confirmed. This study suggests that this negative relationship should be explained in combination with the actual situation of the Poyang Lake ecological economic zone. This zone, which is located in the middle and lower reaches of the Yangtze River, has a subtropical humid monsoon climate with abundant light and rainfall throughout the year. However, excessive sunshine and rainfall might have an excessive effect on the rural population distribution in the region. First, excessive amount of sunshine and rainfall will produce heat and water stresses on local rice cultivation [32, 33]. The Poyang Lake ecological economic zone is located in a typical flood area. Therefore, excessive rainfall during rice pollination stage will affect rice pollination and fruiting and increase the risk of natural disasters, such as flood and waterlogging. These factors will lead to the decline in rice yield per unit. Agricultural development is positively related to the distribution of rural population. Second, excessive levels of sunshine and rainfall are not conducive to the survival and development of rural population. The sumer heat in the Poyang Lake ecological economic zone is difficult to bear. Coupled with global warming and heat, excessive sunshine will increase the temperatures; elevated temperature is not conducive to the construction of rural ecology. As previously mentioned, excessive rainfall increases the risk of natural disasters, such as floods, and endangers the lives and property of the rural population. The density of rural population decreases with the increase in the levels of sunshine and rainfall. The results show that the elasticity of sunshine and rainfall is far beyond those other factors, thereby highlighting that the negative influence of sunshine and rainfall can not be ignored. In addition, the effect of average wind speed on the distribution of regional rural population is significantly positive, because the Poyang Lake ecological economic zone is hot in summer, the wind speed can reduce the human somatosensory temperature to a certain extent.

## Research conclusions and implications

In this study, the county scale is used as a basic unit to analyze the distribution of rural population in the Poyang Lake ecological economic zone from a climate perspective. The spatial autocorrelation method is used to reveal the distribution of rural population and its spatial correlation with the climatic factors. On this basis, the optimal model (SEM) is determined, and the influencing factors and degree of rural population distribution are obtained. The following conclusions are drawn from the results. (1) Rural population is a vulnerable group to deal with climate change, and the influence of climate factors on the distribution of rural population in lake area is far beyond economic factors. Because the population distribution framework and pattern were first determined by climate, topography and other factors, and this kind of population regional structure is relatively stable, although the current economic factors gradually become the dominant factor of population distribution, but in the rural areas with relatively backward economy, this pattern of population regional distribution still has certain stability. Therefore, in the planning and layout of rural population, we should take more account of the influence of natural climate factors, attach great importance to the vulnerability, sensitivity and high impact of climate on the distribution of rural population, deal with the distribution of rural population and urban population differently, fully consider the difference of spatial difference and geographical cost, design the top level of population distribution according to local conditions, and realize the coordinated development of regional population, economy and environment. (2) There are significant negative effects of sunshine and rainfall on rural population distribution in Poyang Lake Ecological Economic Zone, which is contrary to the general law. It shows that geographical capital and natural resource endowment are different, and the influence of climate on the population distribution in micro-region may be different from the general law, which is also the concrete embodiment of geographical difference and law. (3) In the study of climate factors on the distribution of rural population in lake areas, there is a significant spatial correlation between climate factors and rural population distribution. The traditional linear regression model ignores this spatial correlation and easily leads to distortion of the research results. The spatial measurement model which takes spatial dependence into account can reveal the influence of climate on rural population distribution more accurately.

However, this study does not consider the temporal variation of population distribution. In addition, the number of research samples is relatively sall. The future study will expand the sample size, focus on the spatial and temporal differences of rural population distribution, explain the law of rural population distribution and climate change from the perspective of timing, and provide microsupport for rural revitalization, poverty alleviation, and ecological livable construction.

## Supporting information

S1 File (7Z)
